# Low‐grade systemic inflammation biomarkers in sedentary young healthy adults are not significantly affected by a 24‐week concurrent training intervention

**DOI:** 10.1111/nyas.15329

**Published:** 2025-04-12

**Authors:** Héctor Vázquez‐Lorente, Lourdes Herrera‐Quintana, Francisco M. Acosta, Francisco J. Amaro‐Gahete, Jonatan R. Ruiz

**Affiliations:** ^1^ Faculty of Medicine, Department of Physiology University of Granada Granada Spain; ^2^ School of Health Sciences International University of La Rioja Logroño Spain; ^3^ Turku PET Centre University of Turku Turku Finland; ^4^ Turku PET Centre Turku University Hospital Turku Finland; ^5^ InFLAMES Research Flagship Center University of Turku Turku Finland; ^6^ MediCity/PET Preclinical Laboratory University of Turku, Turku PET Centre Turku Finland; ^7^ Faculty of Sport Sciences, Department of Physical Education and Sports University of Granada Granada Spain; ^8^ Centro de Investigación Biomédica en Red Fisiopatología de la Obesidad y Nutrición (CIBERobn) Instituto de Salud Carlos III Madrid Spain; ^9^ Instituto de Investigación Biosanitaria ibs.Granada Granada Spain; ^10^ Sport and Health University Research Institute (iMUDS) University of Granada Granada Spain

**Keywords:** body composition, concurrent training, inflammation, interleukins, low‐grade systemic inflammation

## Abstract

In this study, we measured the dose–response effect of a 24‐week concurrent training (CT) intervention on low‐grade systemic inflammation biomarkers in sedentary young healthy adults. A total of 100 untrained participants were randomized to (1) no exercise (control group, *n* = 35), (2) aerobic + resistance exercise (CT) at moderate intensity (exercise‐moderate group; *n* = 33), or (3) CT at vigorous intensity (exercise‐vigorous group, *n* = 32). Serum concentrations of C‐reactive protein (CRP), interleukin‐6 (IL‐6), IL‐7, IL‐8, and IL‐10, interferon‐gamma (IFN‐γ), tumor necrosis factor‐α, leptin, and adiponectin were determined and compared among the three groups. The exercise‐vigorous group members had lower differences in IL‐7 levels among them, compared to the exercise‐moderate group members (Δ = ‒7.97% vs. 1.90%; *p* = 0.030; 95% CI [‒0.90, ‒0.04]). The exercise‐vigorous group members showed higher differences in CRP values (Δ = 20.1%; *F* = 3.339; *p* = 0.046) compared to both the control (Δ = ‒1.91%) and the exercise‐moderate (Δ = ‒23.3%) group members, whereas the control group exhibited higher differences in IFN‐γ levels compared to the exercise‐vigorous group (Δ = 15.3% vs. 2.62%; *p* = 0.048; 95% CI [‒0.68, ‒0.01]). For individuals in the three groups, body composition and physical fitness correlated overall with leptin. The data show, and we concluded, that the training intervention had no significant effect on low‐grade systemic inflammation biomarkers.

## Graphical abstract

A 24‐week supervised concurrent training intervention in sedentary young healthy adults showed minimal effects on low‐grade systemic inflammation biomarkers. Vigorous exercise reduced interleukin‐7 but increased C‐reactive protein compared to moderate exercise and control groups. The control group had higher interferon‐gamma levels than the vigorous exercise group. Leptin changes were closely related to improvements in body composition and fitness, suggesting its importance. Exercise intensity had overall limited influence on low‐grade inflammation biomarkers.

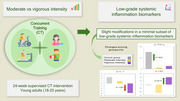

## INTRODUCTION

The prevalence of physical inactivity has dramatically increased in recent decades, especially among younger age groups,^1^ together with a notable rise in the incidence of cardiometabolic disturbances in this population over the same time period.^2^ Physical inactivity is a significant health concern since, among other reasons, it worsens body composition and, consequently, activates a network of inflammatory pathways^3^ favoring low‐grade systemic inflammation that increases the susceptibility to numerous noncommunicable diseases.^4^, ^5^

Low‐grade systemic inflammation is characterized by a persistent increase in circulating blood inflammatory cytokines and acute‐phase proteins.^6^ Among the key mediators, tumor necrosis factor‐α (TNF‐α), C‐reactive protein (CRP), interleukin‐6 (IL‐6), IL‐8, and IL‐10, are among the most commonly used blood biomarkers to characterize low‐grade systemic inflammation.^7^, ^8^ Understanding how these pro‐ and anti‐inflammatory signals are modulated is crucial for developing strategies to mitigate low‐grade systemic inflammation.^4^

Aerobic and resistance training have been suggested to mitigate low‐grade systemic inflammation.^9^ Despite that aerobic training seems to be more effective for that purpose,^10^ as T cells in skeletal muscle shield mitochondria from the effect of interferon‐gamma (IFN‐γ),^11^ their combination (i.e., aerobic + resistance, known as concurrent training [CT]) may be a potential strategy to obtain a synergistic effect.^12^ Potential mechanisms include a reduction in visceral adipose tissue (VAT) mass, increased production and release of muscle‐derived myokines,^13^ decreased cytokine production by endothelial cells, and a decrease of proinflammatory cells and reduced proinflammatory cytokine cell production.^14^ Studies performed in a wide variety of both healthy and unhealthy aged populations found a decrease in the concentrations of multiple low‐grade systemic inflammatory blood biomarkers (mainly TNF‐α, IL‐6, and CRP) following a CT intervention,^13^, ^14^, ^15^, ^16^ while others reported no such effect.^17^, ^18^, ^19^, ^20^, ^21^ Of note, limited research has examined the effect of CT programs on low‐grade systemic inflammation biomarkers in young adults, with most studies reporting inconsistent findings or a lack of effects on the majority of blood inflammatory markers assessed.^22^, ^23^ Indeed, whether these potential effects are dependent on exercise intensity remain unknown at present.^24^ The heterogeneity of human trials, exemplified by variations in study populations, intervention durations, time points when the biomarkers were measured after the intervention, and disparities in exercise modalities or training intensities, may serve as an explanation for the discrepancies observed.^19^, ^25^ Hence, understanding the dose–response effects of CT on low‐grade systemic inflammation biomarkers and selecting the most suitable exercise intervention program for this purpose is needed.^26^

An unfavorable body composition profile, such as a higher accumulation of VAT mass, is related to dysregulation of adipose tissue metabolism and a higher secretion of pro‐inflammatory adipokines, which have been also associated with low‐grade systemic inflammation.^27^, ^28^, ^29^ In contrast, it is known that the skeletal muscle stimulates the release of several cytokines (e.g., IL‐6, IL‐13, and IL‐15) during acute exercise, which in the long term could potentially contribute to reduced low‐grade systemic inflammation by promoting anti‐inflammatory effects.^30^ Moreover, subjects with better physical fitness exhibit lower low‐grade systemic inflammation, partially explained by body composition improvements that could affect the outcome of an intervention targeting systemic inflammation.^10^ We have previously observed that a well‐designed 24‐week supervised CT intervention improves body composition (i.e., fat mass [FM] and VAT mass) and physical fitness independently of intensity in a cohort of sedentary young healthy adults.^31^, ^32^ Nevertheless, whether these dose–response, exercise‐induced enhancements on body composition and physical fitness are related to potential changes in low‐grade systemic inflammation biomarkers remains unknown.

This study investigated the dose–response effect of a 24‐week CT supervised intervention on low‐grade systemic inflammation biomarkers and explored the relationship of exercise‐induced changes in body composition and physical fitness, with low‐grade systemic inflammation biomarkers in sedentary young healthy adults.

## MATERIALS AND METHODS

### Study design

The current study constitutes a secondary analysis of the Activating Brown Adipose Tissue through Exercise (ACTIBATE) trial (the ACTIBATE study; ClinicalTrials.gov ID: NCT02365129)^33^ that originally aimed to investigate the dose–response effect of a 24‐week supervised CT intervention on the mass and activity of brown adipose tissue in young adults.^34^ The intervention was conducted according to the Consolidated Standards of Reporting Trials (CONSORT) guidelines (EQUATOR Network: http://www.equator‐network.org/reporting‐guidelines/consort/). Ethical approval for the study was obtained from the University of Granada Ethics Committee on Human Research (no. 924) and the Servicio Andaluz de Salud (Centro de Granada, CEI‐Granada, Spain) [0838‐N‐2017]. The research adhered to the most recent version of the Declaration of Helsinki (2013 revision),^35^ and all participants provided written informed consent before their inclusion.

### Participants

A total of 100 sedentary young adults (*n* = 67 women) were recruited to participate in the ACTIBATE trial. The study protocol and the description of procedures are provided elsewhere.^33^ The inclusion criteria were as follows: (1) age between 18 and 25 years, (2) a body mass index (BMI) ranging from 18.5 to 35 kg/m^2^, (3) self‐reported sedentary lifestyle (i.e., performing a maximum of 20 min of moderate‐to‐vigorous physical activity per day on less than 3 days per week), (4) stable body weight over the past 3 months, with fluctuations of less than 3 kg, (5) medical clearance for participation in an exercise intervention, (6) absence of medication use for chronic diseases, (7) nonsmoker status, and (8) provision of written informed consent.

**FIGURE 1:**
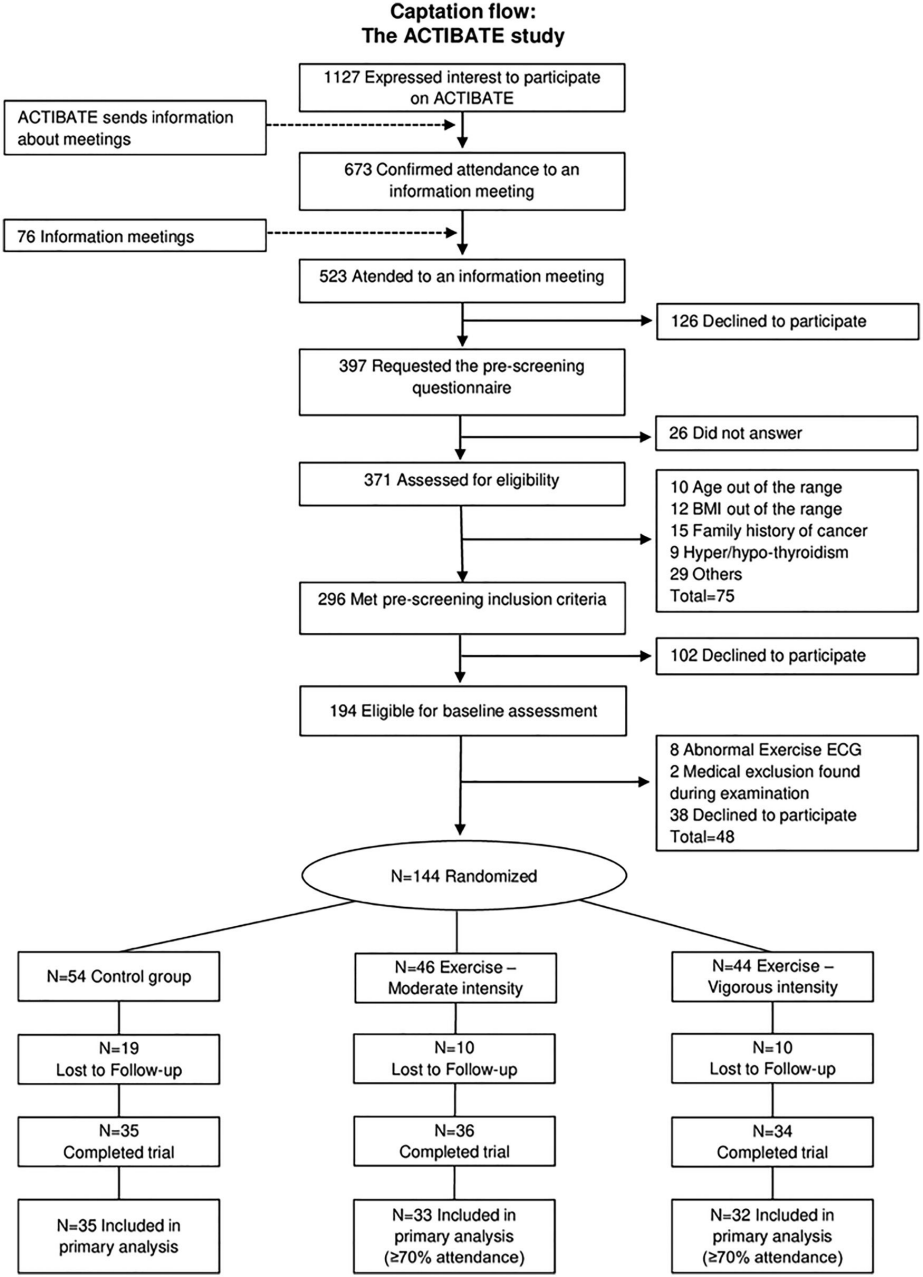
Participant enrollment in the ACTIBATE study. Abbreviations: ACTIBATE, activating brown adipose tissue through exercise; BMI, body mass index; ECG, electrocardiogram.

A 24‐week supervised CT intervention was conducted. After finishing the baseline assessments, participants were randomly allocated to one of three groups through computer‐generated simple unrestricted randomization:^36^ (1) a Control group (*n* = 35) involving no exercise intervention, (2) a CT program at moderate intensity (Ex‐Moderate group, *n* = 33), (3) or an exercise program with similar structure but at vigorous intensity (Ex‐Vigorous group, *n* = 32). A flow‐chart of the participants enrolled in the ACTIBATE study is presented in Figure 1.

### Intervention and training protocol

This study consisted of a 24‐week supervised CT intervention. All participants were explicitly instructed to maintain their regular routine, including physical activity and dietary patterns throughout the study period to ensure consistency in physical activity levels and diet. The 24‐week supervised CT program employed a combination of aerobic and resistance training, aligning with the minimum physical activity recommendations outlined by the World Health Organization (WHO) guidelines.^37^ Certified trainers led sessions in small groups (10–12 people), ensuring personalized attention. The sessions occurred at consistent times over the 24‐week intervention period (either 8:30–10:30 a.m., 4–6 p.m., 6–8 p.m., or 8–10 p.m.). The exercise program comprised five phases with different durations, starting with a 4‐week familiarization phase. Each session began with a dynamic standardized warm‐up (∼10 min) involving joint mobility and compensatory tasks, and concluded with a cool‐down phase (∼10 min) which was based on active global stretching. Continuous monitoring of heart rate (HR) was implemented throughout to control exercise intensity. Sedentary time was assessed through accelerometry using triaxial accelerometer (ActiGraph GT3X+) that participants wore on their nondominant wrist for 1 week.^38^

The participants randomly assigned to the Control group received verbal information about healthy habits including the international physical activity recommendations,^39^ and also nutritional advice based on the Mediterranean diet patterns.^40^ Participants attended the research center for training over a 24‐week period and performed 150 min per week of aerobic training, distributed over three to four sessions per week, plus 80 min per week of resistance training over two sessions per week. Rigorous control over training session attendance was maintained, with electronic attendance sheets used for registration. To ensure compliance, missed sessions were rescheduled on an alternative day. A minimum attendance rate of 70% was set as a benchmark to evaluate the effectiveness of the exercise training intervention. In special circumstances such as holidays or when retaking sessions at the research center was impractical, participants were equipped with a pulsometer, an elastic band, and specific instructions for conducting adapted training sessions at home. Throughout the intervention, participants were encouraged to reach out to the research staff for any queries or concerns. A total of 92% of participants complied with the minimum attendance rate.

The aerobic and resistance training regimens were customized according to each participant's physical fitness level. To ensure an equitable weekly dose for all participants, the duration of the training sessions was adjusted. Programmed sessions had a consistent duration across all experimental groups, independent of the type of intervention. Training loads were tailored to individual fitness levels, and a systematic progression plan was implemented for each exercise training program. Both interventions were time‐matched but programmed with different intensities and, consequently, different caloric dose. Aerobic training sessions included the use of a treadmill, stationary bike, or elliptical ergometer organized in blocks of 10 min, with a short break between them, all performed at the predetermined intensity. In the Ex‐Moderate group, aerobic exercises consisted of a total of 150 min per week of aerobic exercise at an intensity of 60% of heart rate reserve (HRres), whereas the Ex‐Vigorous group engaged in 75 min per week at moderate intensity (60% HRres) and 75 min per week at vigorous intensity (80% HRres). The calculation of HRres, determined as the difference between resting (assessed lying in a quiet, mildly lit room, with controlled environmental conditions in the morning after an overnight fast and avoiding any moderate or vigorous physical activity before the test day for 24 and 48 h, respectively) and maximum values (during a maximum effort test on a treadmill), remained constant throughout the intervention without reassessment. The Ex‐Vigorous group underwent a progressive increase in aerobic exercise intensity during the familiarization period until reaching the target intensity.^33^ Adherence to the prescribed intensity was monitored using HR monitors (RS800CX, Polar Electro Öy) during exercise sessions to ensure consistency. On the other hand, the resistance training sessions comprised a set of 8–9 comprehensive global resistance exercises involving two sets of 10 repetitions each. Weight‐bearing and guided pneumatic machines were used to perform them, targeting both upper and lower body muscle groups. The included exercises were Romanian deadlift, bench press, lateral pull‐down, squat, deadlift, and hip thrust, among others. Additionally, compensatory exercises (e.g., flexibility and core stability exercises) were prescribed to mitigate injury risk and enhance participant adherence. For the Ex‐Moderate group, resistance exercise was set at 50% of the one‐repetition maximum (1‐RM), while the Ex‐Vigorous group performed at 70% of 1‐RM. Reassessment of 1‐RM occurred every 5 weeks to facilitate an effective progression in resistance training. A comprehensive description of this program has been previously published, and additional details of the CT intervention can be found elsewhere.^33^

### Outcomes

At 1–3 weeks before starting the exercise intervention, and at 3–4 days after the last exercise session, blood samples were drawn in the morning following an overnight fast, refraining from physical activity, and consuming a standardized dinner the day before. Afterwards, these samples were centrifuged, and serum and plasma were aliquoted and stored at −80°C for future analyses. All blood samples were obtained at the same time of day before and after the intervention.

### Blood inflammatory markers

Serum CRP (mg/L) concentrations were measured by immunoturbidimetric assay using an AU5832 automated analyzer (Beckman Coulter Inc.). Plasma IL‐6, IL‐7, IL‐8, IL‐10, IFN‐γ, and TNF‐α—all expressed in picograms per milliliter—were determined using the MILLIPLEX MAP Human High Sensitivity Cytokine Panel from the Luminex Corporation (Luminex Corp.; Catalogue #HSCYTMAG‐28SK). Plasma leptin (µg/L) and adiponectin concentrations (mg/L) were measured using the MILLIPLEX MAG Human Adipokine Magnetic Bead Panel 2 kit (Catalogue #HADK2MAG‐61K) and the MILLIPLEX MAP Human Adipokine Magnetic Bead Panel 1 kit (Catalogue #HADK1MAG‐61K), respectively, both from the Luminex Corporation. Using the results from a subsample of the present subjects, intra‐assay coefficients of variation (CVs) were calculated for the set of cytokines studied: IL‐6 = 7.7%, IL‐7 = 7.3%, IL‐8 = 6.6%, IL‐10 = 20.7%, IFN‐γ = 10.3%, and TNF‐α = 8.3%. The CVs for leptin and adiponectin were 9% and 7.8%, respectively.

### Anthropometry and body composition

Participants’ weight (kg) and height (m) were measured using a model 769 calibrated digital scale (Electronic Column Scale) and an analog portable model 213 stadiometer (SECA) with a precision of 0.1 kg and 0.1 cm, respectively. BMI was calculated as body weight (kg)/height^2^ (m^2^). Body composition outcomes, that is, FM (expressed in kg and %), lean mass (LM) (kg), and VAT mass (g), were determined by a whole‐body dual‐energy X‐ray absorptiometry scanner using a Discovery Wi device (DXA; Hologic Wi, Hologic Inc.). The fat mass index (FMI) was calculated as FM (kg)/height^2^ (m^2^) and the lean mass index (LMI) was obtained as LM (kg)/height^2^ (m^2^).

### Physical fitness

Physical fitness was assessed in two different sessions, one focusing on muscular strength and the other on cardiorespiratory fitness.^33^ Before each session, participants fasted for 3–5 h and performed either no vigorous exercise in the previous 48 h or moderate exercise in the previous 24 h, and did not consume caffeine‐containing beverages the day of the tests.^33^

#### Muscle strength

Handgrip strength (measured in kg) was evaluated using a digital hand dynamometer (T.K.K. 5401 Grip‐D; Takey).^41^ Participants performed two maximal‐effort trials per hand, holding the contraction for approximately 3 s, with a 1‐min rest between attempts. The grip span was set to 5.5 cm for men, while for women, a validated equation based on hand size was used to determine the optimal grip span. The highest recorded values from the left and right hands were added together to determine total handgrip strength. For upper and lower body strength, the 1‐RM in the leg press and bench press exercises was estimated using Keiser Sports Health Equipment and the Wathen equation and expressed in kilograms.^42^ A submaximal protocol was applied, starting with a warm‐up of 15 repetitions at roughly 50% of the estimated 1‐RM. The assessment team then selected a load intended to induce muscular failure within fewer than 10 repetitions. If participants completed at least one but fewer than 10 repetitions, the result was considered valid for further analysis. Up to three attempts were permitted, with 3‐min rest intervals between them.^33^

#### Cardiorespiratory fitness

Cardiorespiratory fitness was assessed through a maximal graded treadmill test (H/P/Cosmos Pulsar treadmill, H/P/Cosmos Sport & Medical GMBH) using the modified Balke protocol.^33^ The test began with a warm‐up phase: walking at 3.5 km/h for 1 min, followed by 2 min at 4.0 km/h. After the warm‐up, participants walked at 5.3 km/h with a 0% incline for 1 min, then the incline increased by 1% every minute until the participant reached voluntary exhaustion. Gas exchange was continuously monitored using indirect calorimetry with an oronasal mask (model 7400; Hans Rudolph Inc.) and a preVent™ high‐flow sensor (Medgraphics Corp.). Daily flow calibration was performed using a 3‐L syringe, and gas analyzers were calibrated prior to each test with two standard gas mixtures following the manufacturer's guidelines. VO_2_ and VCO_2_ values were averaged every 5 s using Breeze Suite software (version 8.1.0.54 SP7, MGC Diagnostic; Medgraphics Corp.). HR (beats/min) was recorded continuously at 5 s intervals using a Polar RS800CX HR monitor paired with an H3 chest strap sensor. The rating of perceived exertion (RPE‐CR10) was collected during the final 15 s of each stage and at the point of exhaustion. The time to exhaustion (s) was obtained in a maximum effort test. The VO_2_max criteria included^43^ (1) a change in VO_2_ <100 mL/min during the final 30 s, (2) achieving a respiratory exchange ratio ≥1.1, and (3) reaching a HR within ±10 beats/min of the predicted maximum. When these criteria were not met, peak oxygen uptake was calculated instead. In case of disagreement between evaluators, a third researcher provided an additional opinion. VO_2_max was expressed both in absolute terms (mL/min) and relative to body weight (mL/kg/min).

### Statistical analysis

The sample size calculation for the main outcome is documented elsewhere.^34^ Since this study is a secondary analysis of the ACTIBATE trial and involves exploratory analysis, formal a priori calculations of statistical power were not conducted.

The normality of the data was assessed by the Shapiro–Wilk test, visual histograms, and Q–Q plots. None of the body composition, physical fitness, and low‐grade systemic inflammation biomarkers exhibited normal distribution. Therefore, their values were log10 transformed when using parametric analyses. Delta values (Δ: post‐baseline values) were computed for each outcome.

Data are expressed as mean ± standard deviation, unless otherwise stated. Analysis of variance (ANOVA) was conducted with Bonferroni post hoc adjustments for multiple comparisons to examine the baseline differences among the three groups. A repeated‐measures ANOVA was conducted to examine changes in blood inflammatory markers across time, between groups, and the interaction effect (time × group). An analysis of covariance was performed to study the change observed across the groups (fixed factor) on the blood inflammatory markers (e.g., post‐leptin minus pre‐leptin [dependent variable]), adjusting for the baseline low‐grade systemic inflammation biomarker values. The previously mentioned analyses were additionally adjusted for sex, sedentary time, or limiting the study cohort to individuals with a BMI ≥ 25. To conduct pairwise comparisons between groups, we applied the Bonferroni post hoc test with adjustment for multiple comparisons. Eta squared (η^2^) effect size was considered (small effect: η^2^ = 0.01–0.05; medium effect: η^2^ = 0.06–0.13; large effect: η^2^ ≥ 0.14).^44^ Spearman correlations were performed to analyze the association between body composition and low‐grade systemic inflammation biomarkers and between physical fitness and low‐grade systemic inflammation biomarkers at baseline and over time.

All statistical analyses were performed using the Statistical Package for the Social Sciences v.28.0 (IBM Corporation). All graphs were plotted using GraphPad Prism software v.9.0 (GraphPad Software). Significance was set at *p* ≤ 0.05.

## RESULTS

The baseline characteristics of the study's participants are presented in Table 1. All variables were similar across the three experimental groups except for TNF‐α, whose levels were significantly higher in the Ex‐Vigorous group (*p* = 0.039) compared to the Control and Ex‐Moderate groups. Baseline associations of body composition and physical fitness with low‐grade systemic inflammation biomarkers are shown in Tables S1 and S2, respectively. The effect of the 24‐week supervised CT intervention on body composition and physical fitness has been previously reported.^31^, ^32^

**TABLE 1:** Descriptive characteristics of the study participants included in the per‐protocol analysis at baseline.

	All (*n* = 100)		Control (*n* = 35)		Ex‐Moderate (*n* = 33)		Ex‐Vigorous (*n* = 32)		*p*‐Value
	Mean	SD	Mean	SD	Mean	SD	Mean	SD	
Demographics									
Age (years old)	22.2	2.2	22.0	2.1	22.1	2.2	22.4	2.5	0.732
Male (*n*/%)	33	33.0	14	40.0	9	27.3	10	31.3	–
Female (*n*/%)	67	67.0	21	60.0	24	72.7	22	68.7	–
Body composition									
BMI (kg/m^2^)	24.6	4.1	24.1	3.9	24.9	4.2	24.9	4.1	0.458
LMI (kg/m^2^)	15.1	2.3	14.9	2.5	15.2	2.0	15.4	2.5	0.961
Body fat (%)	33.5	7.5	33.6	7.2	33.8	8.4	33.1	7.1	0.388
FMI (kg/m^2^)	8.2	2.8	8.1	2.7	8.4	3.2	8.2	2.4	0.306
VAT mass (g)	311.4	164.5	310.3	166.1	314.1	177.7	309.7	153.7	0.561
Physical fitness									
Hand grip strength (kg)	31.1	7.5	31.7	7.1	30.6	7.7	31.1	7.9	0.793
1‐RM leg press (kg)	200.3	61.8	203.5	62.0	193.3	57.7	204.9	67.3	0.813
1‐RM bench press (kg)	30.9	12.7	32.7	14.7	28.6	10.5	31.7	13.0	0.628
VO_2_max (mL/min)	2880.9	794.3	2919.1	880.0	2827.8	538.2	2896.7	937.4	0.959
VO_2_max (mL/kg_weight_/min)	41.2	8.5	42.7	9.6	40.5	6.7	40.3	9.0	0.580
Time to exhaustion (s)	937.8	193.5	926.1	210.4	938.4	191.0	950.8	181.9	0.830
HRmax (beats/min)	194.6	10.5	193.6	10.8	193.8	8.9	196.3	11.6	0.569
Low‐grade systemic inflammation biomarkers									
IL‐6 (pg/mL)	2.9	2.8	2.8	2.8	2.7	2.8	3.3	3.0	0.880
IL‐7 (pg/mL)	7.6	5.3	7.7	4.9	7.0	5.7	8.1	5.5	0.550
IL‐8 (pg/mL)	2.9	1.5	2.9	1.5	2.8	1.4	3.0	1.6	0.874
IL‐10 (pg/mL)	5.8	7.3	6.0	9.7	6.0	6.9	5.3	4.2	0.774
CRP (mg/L)	2.5	3.5	2.1	2.2	3.5	5.2	1.9	2.2	0.417
IFN‐γ (pg/mL)	26.1	10.7	23.7	10.4	27.9	9.5	26.9	11.9	0.221
TNF‐α (pg/mL)	3.5	2.2	3.1	1.7	3.2	1.4	4.3	3.1	**0.039**
Adiponectin (mg/L)	11.2	7.6	11.8	7.0	11.0	8.2	10.7	7.8	0.426
Leptin (µg/L)	6.1	4.4	5.4	4.5	6.8	4.7	6.3	4.0	0.509

Data are presented as mean and SD. All variables were log10 transformed before further analyses. One‐way ANOVA was used for comparing differences between groups. Statistical significance was defined as *p*‐value <0.05. Abbreviations: 1‐RM, one‐repetition maximum; BMI, body mass index; CRP, C‐reactive protein; Ex‐Moderate, moderate intensity group; Ex‐Vigorous, vigorous intensity group; FMI, fat mass index; HRmax, maximal heart rate; IFN‐ɣ, interferon‐gamma; IL, interleukin; LMI, lean mass index; SD, standard deviation; TNF‐α, tumor necrosis factor‐alpha; VAT, visceral adipose tissue; VO_2_max, maximal oxygen uptake.

**FIGURE 2:**
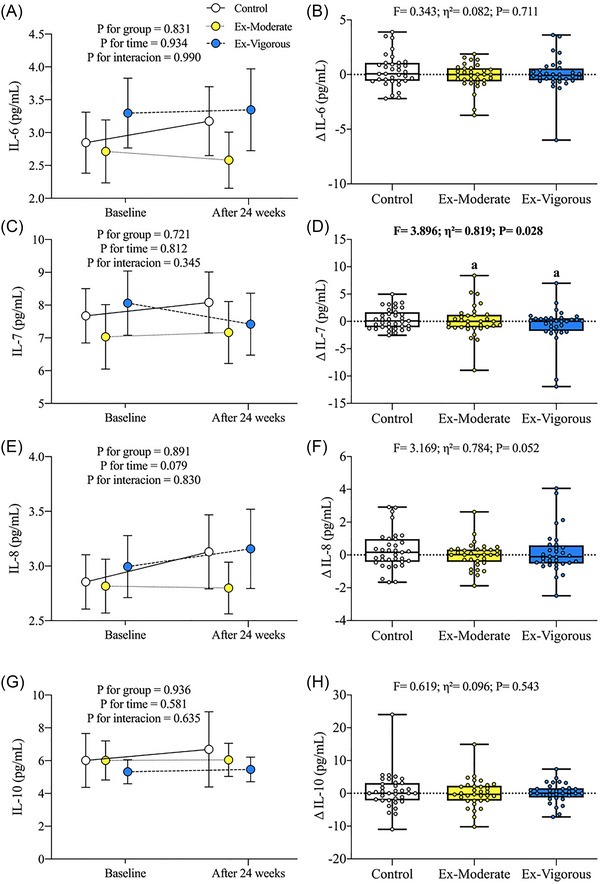
Changes in plasma interleukins after the 24‐week intervention. Data are expressed as mean ± standard deviations. All interleukin values were log10 transformed before further analyses. Δ represents changes in interleukins after intervention. Statistical significance was defined as *p*‐value <0.05. *p*‐Values (time, group, and interaction [time × group]) of repeated‐measures ANOVA (A, C, E, G). *p*‐Value of analysis of covariance (ANCOVA) adjusting for baseline interleukin values (B, D, F, H). Post hoc Bonferroni corrections were performed for pairwise comparisons. Eta squared (η^2^) effect size (small effect: η^2^ = 0.01–0.05; medium effect: η^2^ = 0.06–0.13; large effect: η^2^ ≥ 0.14). Abbreviations: Ex‐Moderate, moderate intensity group; Ex‐Vigorous, vigorous intensity group; IL = interleukin.

The effect of the 24‐week supervised CT intervention on ILs can be found in Figure 2. No significant time × group interaction effect was revealed after the intervention for any studied ILs (Figure 2A,C,E,G). The Ex‐Vigorous group showed lower differences in IL‐7 levels compared to the Ex‐Moderate group (Δ = ‒7.97% vs. 1.90%; *p* = 0.030; 95% CI [‒0.90, ‒0.04]; Figure 2D), while no significant differences were noted in the other ILs after the intervention across groups (Figure 2B,F,H). All the previously mentioned results persisted after including sex and sedentary time as a covariate or limiting the study cohort to individuals with a BMI ≥ 25 (data not shown).

**FIGURE 3:**
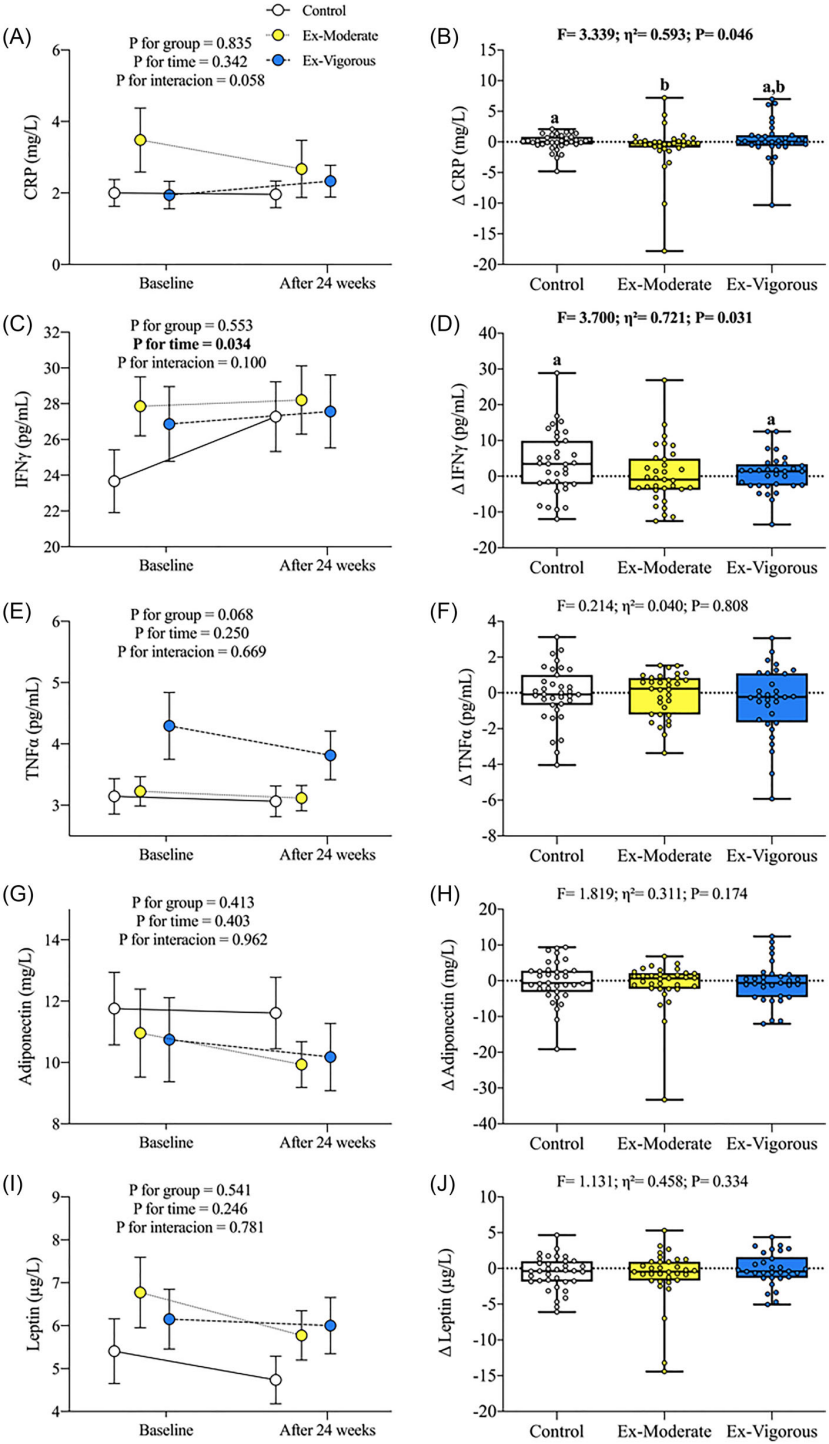
Changes in other low‐grade systemic inflammation biomarkers after the 24‐week intervention. Data are expressed as mean ± standard deviations. All variables were log10 transformed before further analyses. Δ represents changes in low‐grade systemic inflammation biomarkers after intervention. Statistical significance was defined as *p*‐value <0.05. *p*‐Values (time, group, and interaction [time × group]) of repeated‐measures ANOVA (A, C, E, G, I). *p*‐Value of analysis of covariance (ANCOVA) with post hoc Bonferroni‐corrected (similar letters indicate significant differences) for the change in the low‐grade systemic inflammation variable adjusting for baseline values (B, D, F, H, J). Eta squared (η^2^) effect size (small effect: η^2^ = 0.01–0.05; medium effect: η^2^ = 0.06–0.13; large effect: η^2^ ≥ 0.14). Abbreviations: CRP, C‐reactive protein; Ex‐Moderate, moderate intensity group; Ex‐Vigorous, vigorous intensity group; IFN‐γ, interferon‐gamma; TNF‐α, tumor necrosis factor‐alpha.

Figure 3 represents the effects of the 24‐week supervised CT intervention upon other low‐grade systemic inflammation biomarkers. No significant time × group effects were observed in the low‐grade systemic inflammation biomarkers (Figure 3A,C,E,G,I). The Ex‐Vigorous group showed higher differences in CRP values (Δ = 20.1%; *F* = 3.339; *p* = 0.046; Figure 3B) compared to both the Control (Δ = ‒1.91%) and the Ex‐Moderate (Δ = ‒23.3%) groups, whereas the Control group exhibited higher differences in IFN‐γ levels compared to the Ex‐Vigorous group (Δ = 15.3% vs. 2.62%; *p* = 0.048; 95% CI [‒0.68, ‒0.01]; Figure 3D). No significant differences were detected in the rest of the low‐grade systemic inflammatory biomarkers after the intervention across the three groups (Figure 3F,H,J). All the previous mentioned results persisted after adjusting for sex and sedentary time or limiting the study cohort to individuals with a BMI ≥ 25 (data not shown).

**FIGURE 4:**
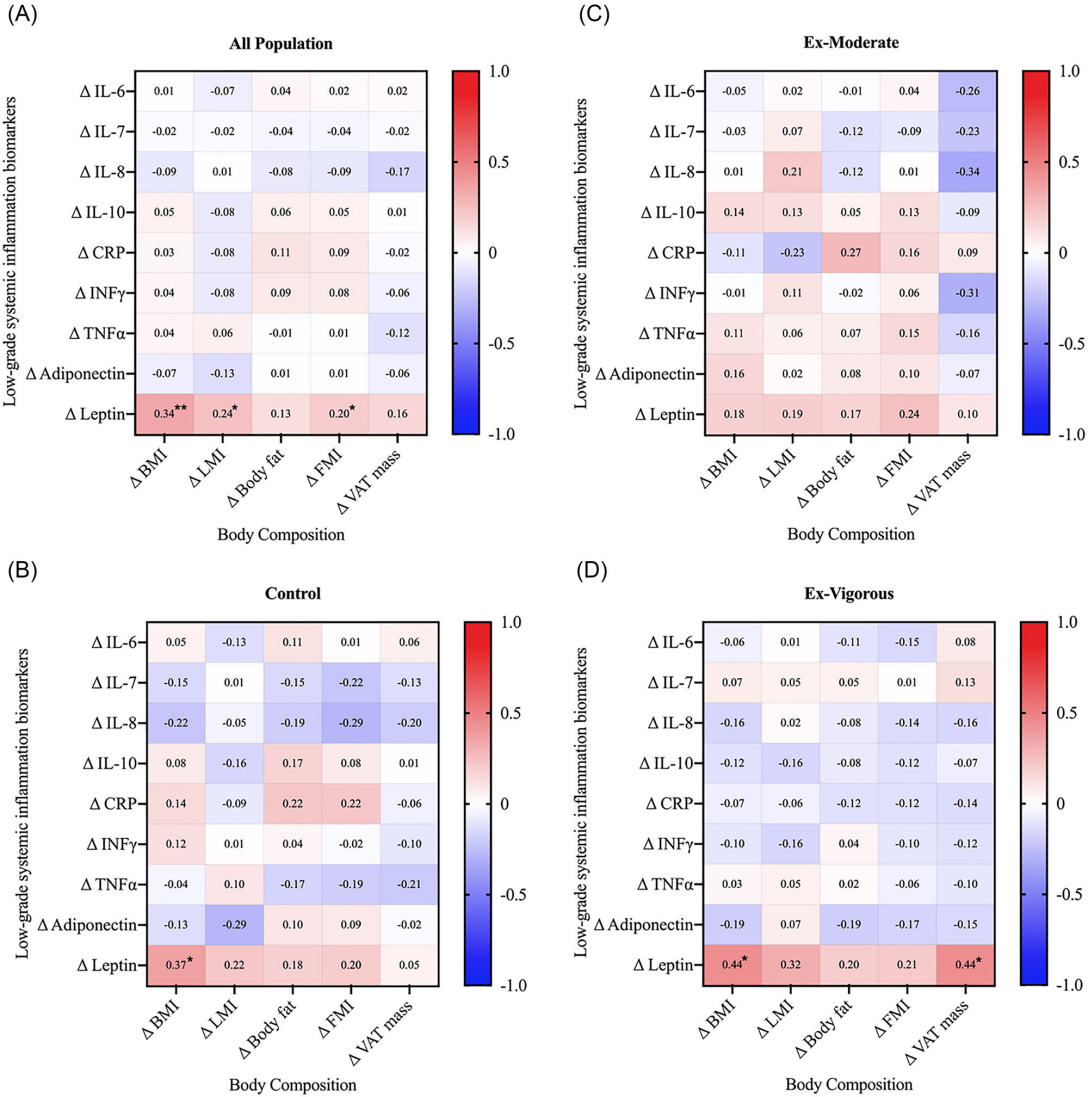
Spearman correlations between changes in body composition and changes in low‐grade systemic inflammation biomarkers in all participants (A) and by intervention groups (B‐D). Matrix correlations are presented as Spearman correlation coefficients (rho). Significance: *p‐value < 0.05, **p‐value < 0.001. Abbreviations: BMI, body mass index; CRP, C‐reactive protein; FMI, fat mass index; IFN‐γ, interferon‐gamma; IL, interleukin; LMI, lean mass index; TNF‐α, tumor necrosis factor‐alpha; VAT, visceral adipose tissue.

Figure 4 presents the relationships between exercise‐induced changes in body composition and low‐grade systemic inflammation biomarkers. Changes in BMI, LMI, and FMI were positively related to those obtained in leptin in all participants (all rho ≥ 0.20; all *p* ≤ 0.017). These results persisted after adjusting for sex and sedentary time or limiting the study cohort to individuals with overweight or obesity (data not shown).

**FIGURE 5:**
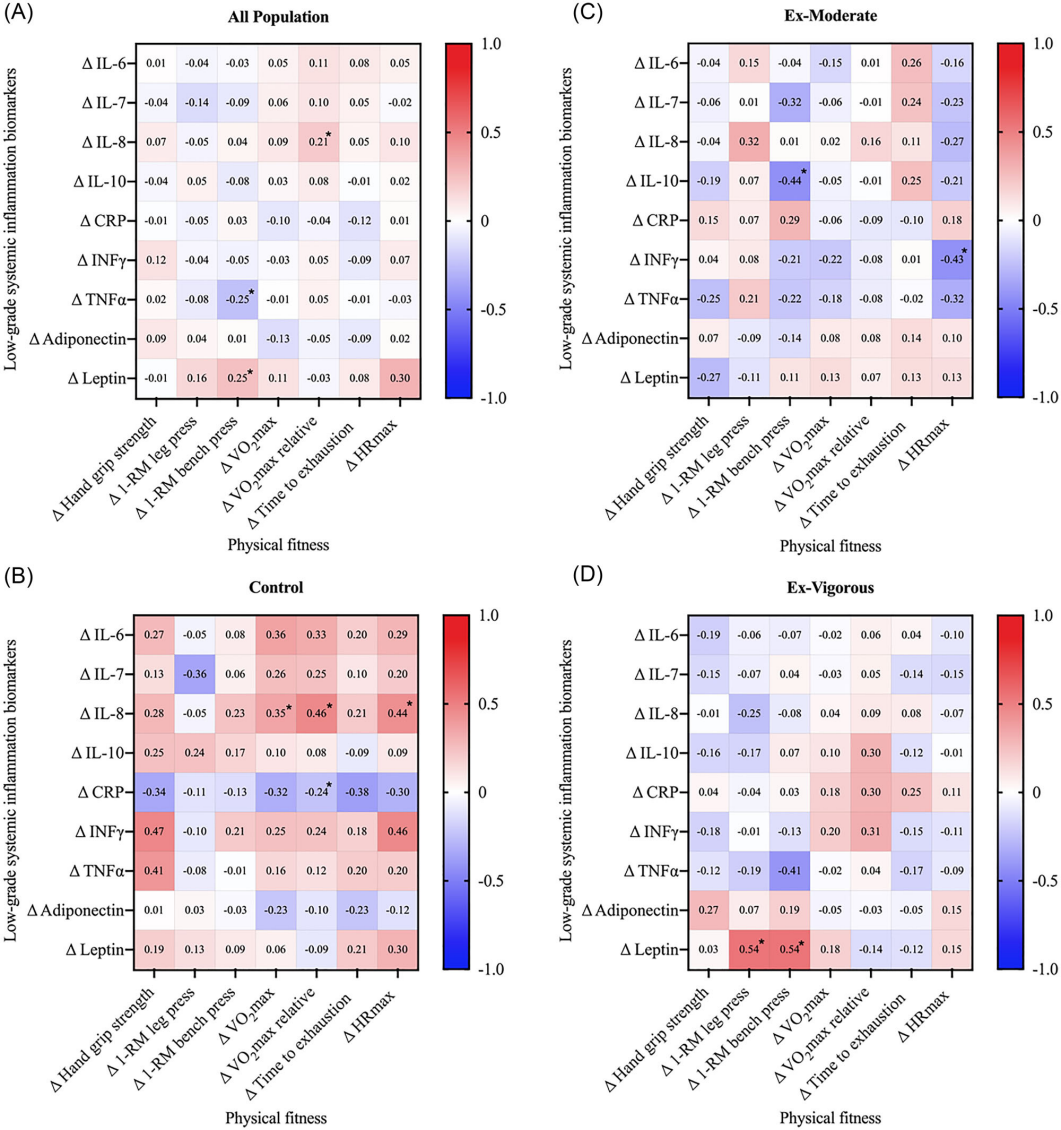
Spearman correlations between changes in physical fitness and changes in low‐grade systemic inflammation biomarkers in all participants (A) and by intervention groups (B‐D). Matrix correlations are presented as Spearman correlation coefficients (rho). Significance: *p‐value <0.05, **p‐value <0.001. Abbreviations: 1‐RM, one‐repetition maximum; BMI, body mass index; CRP, C‐reactive protein; FMI, fat mass index; HRmax, maximal heart rate; IFN‐γ, interferon‐gamma; IL, interleukin; LMI, lean mass index; TNF‐α, tumor necrosis factor‐alpha; VAT, visceral adipose tissue; VO_2_max, maximal oxygen uptake.

Figure 5 shows the correlations between exercise‐induced changes in physical fitness and changes in low‐grade systemic inflammation biomarkers. In all participants, changes in 1‐RM leg press were positively associated with changes in leptin (rho = 0.25; *p* ≤ 0.022) and inversely associated with changes in TNF‐α (rho = ‒0.25; *p* ≤ 0.023). Moreover, changes in VO_2_max were positively associated with changes in IL‐8 (rho = 0.21; *p* = 0.045). The results persisted after adjusting for sex and sedentary time or limiting the study cohort to individuals with overweight and obesity (data not shown).

## DISCUSSION

The present study aimed to investigate the dose–response effects of a 24‐week supervised CT program based on current WHO physical activity guidelines on low‐grade systemic inflammation biomarkers of sedentary young healthy adults. Overall, the findings indicate that regardless of the exercise intensity, the exercise intervention program had no remarkable effect on the low‐grade systemic inflammation biomarkers, as evidenced by the limited number of inflammation biomarkers influenced by the intervention. Overall, alongside leptin, changes in exercise‐induced body composition and physical fitness were not associated with changes in low‐grade systemic inflammation biomarkers. The youthfulness of the participants and their apparent optimal health status characterized by low‐grade systemic inflammation biomarkers within healthy ranges may explain the absence of observed effects.

Over the past 20 years, the regulation of the inflammatory process has been widely investigated to increase its understanding while, at the same time, revealing its complexity due to the large number of extracellular and intracellular mediators, as well as the complex array of signaling pathways.^45^ Exercise induces acute increases in both anti‐inflammatory and proinflammatory ILs, and chronic physical exercise has been associated with long‐term changes in these biomarkers, which may be attributed to the muscle adaptation processes triggered by regular exercise.^46^ Interestingly, we did not observe any of these adjustments in IL levels after our 24‐week supervised CT intervention, data that concur with those observed in a cohort of healthy middle‐aged individuals after 16 weeks of a CT program at moderate‐to‐vigorous intensity.^19^ Studies evaluating the role of chronic physical exercise interventions on a broad range of ILs are scarce and most of them are focused on IL‐6, a widely recognized exerkine.^47^ Given that IL‐6 serves as an indicator of chronic inflammation,^48^ individuals who completed physical exercise interventions and stemming from a nonchronic inflammation status do not usually demonstrate modifications in IL‐6 levels, as evidenced in a study involving women engaged in a 16‐week CT program.^49^ Similarly, no significant differences for circulating blood inflammatory ILs have been reported between young untrained controls versus aged^50^ or young athletes.^51^ Although in general terms we did not find any significant effects of our 24‐week supervised CT intervention on the multiple ILs analyzed, the Ex‐Vigorous group reduced IL‐7 levels compared to the Ex‐Moderate group (Δ = ‒7.97% vs. 1.90%). To date, no clear evidence regarding the role of physical exercise on IL‐7 has been reported.^52^ IL‐7 may inhibit the development of the muscle fiber phenotype.^53^ Interestingly, adaptive changes of skeletal muscle in response to physical activity may include adjustments in the production of IL‐7.^54^ However, it is difficult to know whether changes in IL‐7 were induced by our 24‐week supervised CT intervention. Elucidating the role of IL‐7 on exercise‐induced adaptative processes and its real implications in muscle function is therefore warranted.

Our findings also showed significant changes in CRP and IFN‐γ levels after the 24‐week supervised CT intervention. First, the Ex‐Vigorous group showed higher differences for CRP levels (Δ = 20.1%) compared with both the Control (Δ = ‒1.91%) and the Ex‐Moderate (Δ = ‒23.3%) groups. CRP is considered as one of the most reliable and accurate inflammatory markers since its long‐half life in circulation allows to detect the presence of chronic inflammation.^55^ Moreover, CRP is usually considered as the inflammatory parameter most significantly influenced by exercise over time.^56^ Higher CRP blood concentrations have been previously found when exercising at higher intensities after 6 weeks of CT due to the augmented level of physical stress evoked by the more strenuous exercise protocol.^57^ On the other hand, it must be taken into consideration that CRP may have an acute phase response following exercise, persisting at elevated levels for several days subsequent to the final training session.^58^ Second, the Control group showed higher differences in IFN‐γ levels compared to the Ex‐Vigorous group (Δ = 15.3% vs. 2.62%). A previous study implementing a 16‐week moderate‐to‐high‐intensity CT intervention reported that the program prevented the augmentation of IFN‐γ levels in patients with Alzheimer's disease.^59^ However, no changes of such variable were noted in middle‐aged women with cancer who underwent different CT interventions. Interestingly, and opposite to our findings, a moderate‐to‐high‐intensity 16‐week CT intervention in a cohort of middle‐aged healthy men did not affect CRP and TNF‐α levels regardless of intensity, suggesting that the combination of a healthy inflammatory status and a short intervention period may not be sufficient to produce significant changes in low‐grade systemic inflammation biomarkers.^19^ Nevertheless, 12 weeks of CT at vigorous intensity in middle‐aged overweight or obese men diminished CRP, TNF‐α, and adiponectin levels, suggesting that the CT intervention presents more remarkable effects at improving low‐grade systemic inflammation biomarkers when subjects are unhealthy.^12^ Along this line, two recent systematic reviews examining the effects of chronic exercise training on circulating pro‐ and anti‐inflammatory cytokines concluded that CT is a feasible strategy for enhancing specific inflammatory cytokines but only when it is done by metabolically unhealthy individuals.^4^, ^60^ Furthermore, people who suffer metabolic conditions with a poorer prognosis demonstrated additional improvements in most inflammatory outcomes when engaging in CT protocols.^61^ The lack of effects in our study could be partially explained by the young age of the participants as well as by their relatively healthy status.^62^

Regarding the relationship between changes in body composition, physical fitness, and changes in low‐grade systemic inflammation biomarkers over time, leptin emerged as the biomarker predominantly influenced after our 24‐week supervised CT program, as evidenced in the direct relationships observed for BMI, LMI, FMI, and 1‐RM leg press in all participants. Elevated leptin levels have been noted in younger cohorts, since this hormone is significantly influential in human development.^63^ Leptin is secreted by adipose tissue in direct relation to the amount of FM content,^64^ being the BMI threshold where leptin starts to be considerable increased lower than the cutoff for overweight.^65^ Leptin is thus dependent on the body composition status,^63^ which is modulated by physical fitness.^14^ Moreover, it has been reported that body composition affects the balance of cytokines in subjects with chronic low‐grade inflammation.^66^ In fact, differences in low‐grade systemic inflammation biomarkers of young subjects categorized as normal weight, overweight/obese, and underweight have been previously documented, with greater alterations observed in both below and above the normal weight range.^67^ However, even when limiting our study cohort to overweight individuals, no effect was observed. Further investigation is warranted, given that most studies rely on unhealthy populations.^68^

The primary strength of this study lies in its examination of various intensities of CT, offering potential for tailored exercise programs to address diverse conditions. Nevertheless, our study has some limitations that should be addressed: (1) sample imbalance in sex distribution and (2) limited generalizability of findings to younger or older age groups and trained individuals, as our study exclusively recruited untrained young adults aged 18–25.

## CONCLUSION

Our 24‐week supervised CT intervention showed no overall remarkable effects on low‐grade systemic inflammation biomarkers in sedentary young healthy adults regardless of the exercise intensity. The observed differences in a minimal subset of biomarkers, particularly leptin—one of the most relevant biomarkers of low‐grade systemic inflammation influenced by changes in body composition and physical fitness—may suggest adaptive adjustments in response to exercise. Nevertheless, further investigation is needed to confirm these findings. The complex interplay among these variables across various processes poses challenges in establishing cause–effect relationships. Further research involving different CT protocols is warranted in populations exhibiting different biological characteristics, where the effects of exercise may be more pronounced, potentially leading to changes in low‐grade systemic inflammation biomarkers.

## AUTHOR CONTRIBUTIONS

**H.V.‐L. and L.H.‐Q**.: Conceptualization; formal analysis; software; visualization; writing—original draft. **F.M.A**.: Conceptualization; formal analysis; software; visualization; writing—review and editing. **F.J.A.‐G**.: Conceptualization; data curation; formal analysis; supervision; validation; visualization; writing—review and editing. **J.R.R**.: Conceptualization; methodology; funding acquisition; project administration; resources; supervision; validation; visualization; writing—review and editing. All authors approved the final draft of the manuscript for publication.

## CONFLICT OF INTEREST STATEMENT

The authors declare no conflicts of interest.

### CLINICAL TRIAL REGISTRATION

The ACTIBATE study; ClinicalTrials.gov ID: NCT02365129.

### PEER REVIEW

The peer review history for this article is available at https://publons.com/publon/10.1111/nyas.15329.

## Supporting information



Supplementary Materials.

## Data Availability

These data have not been previously presented anywhere and will be shared upon reasonable request to the corresponding authors, Héctor Vázquez‐Lorente and Jonatan R. Ruiz (hectorvazquez@ugr.es; ruizj@ugr.es).
