# Supplementary material for: Low‐grade systemic inflammation biomarkers in sedentary young healthy adults are not significantly affected by a 24‐week concurrent training intervention
Source: Ann N Y Acad Sci. 2025 Apr 12;1547(1):154–69. doi: 10.1111/nyas.15329 (PMC12096816; doi:10.1111/nyas.15329)
Supplement: Supplementary file 1 — Supplementary Materials. [file NYAS-1547-154-s001.docx]

| **Table S1.-** Association of body composition parameters with low-grade systemic inflammation biomarkers at baseline. | | | | | |
| --- | --- | --- | --- | --- | --- |
|  | BMI | LMI | Body fat | FMI | VAT mass |
| IL-6 | 0.02 | -0.13 | 0.11 | 0.08 | 0.03 |
| IL-7 | -0.18 | **-0.31^**^** | 0.03 | -0.05 | -0.15 |
| IL-8 | 0.02 | -0.17 | 0.18 | 0.14 | 0.03 |
| IL-10 | 0.07 | -0.05 | 0.18 | 0.15 | 0.03 |
| CRP | **0.35^**^** | 0.07 | **0.40^**^** | **0.43^**^** | **0.35^**^** |
| IFNγ | 0.10 | -0.02 | 0.20 | 0.19 | 0.01 |
| TNFα | 0.01 | -0.17 | **0.22^*^** | 0.15 | -0.09 |
| Adiponectin | **-0.28^**^** | **-0.41^**^** | 0.09 | -0.07 | **-0.33^**^** |
| Leptin | **0.47^**^** | -0.05 | **0.74^**^** | **0.72^**^** | **0.41^**^** |
| Matrix correlations are presented as Spearman correlation coefficients (Rho). Significance (boldfaced): *P-value < 0.05, **P-value < 0.001. Abbreviations: BMI = body mass index; CRP = C-reactive protein; FMI = fat mass index; IFNɣ = interferon-gamma; IL = interleukin; LMI = lean mass index; TNFα = tumor necrosis factor-alpha; VAT = visceral adipose tissue. | | | | | |

| **Table S2.-** Association of physical fitness parameters with low-grade systemic inflammation biomarkers at baseline. | | | | | | | |
| --- | --- | --- | --- | --- | --- | --- | --- |
|  | Hand grip strength | 1-RM leg press | 1-RM bench press | VO_2_max | VO_2_max relative | Time to exhaustion | HRmax |
| IL-6 | **-0.24*** | -0.19 | **-0.24*** | -0.14 | -0.04 | -0.01 | -0.12 |
| IL-7 | **-0.26*** | **-0.31*** | **-0.36**** | **-0.24*** | 0.03 | 0.02 | -0.12 |
| IL-8 | **-0.27*** | **-0.27*** | **-0.27*** | -0.15 | -0.06 | -0.01 | -0.01 |
| IL-10 | -0.07 | -0.06 | -0.09 | 0.01 | 0.01 | -0.06 | -0.02 |
| CRP | -0.138 | 0.08 | -0.08 | 0.02 | **-0.34*** | **-0.38**** | 0.12 |
| IFNγ | -0.05 | -0.06 | -0.06 | 0.02 | -0.07 | -0.15 | 0.02 |
| TNFα | -0.21 | **-0.27*** | -0.20 | -0.18 | -0.15 | -0.18 | -0.04 |
| Adiponectin | -0.2 | **-0.45***** | **-0.37**** | **-0.27*** | 0.01 | -0.05 | 0.01 |
| Leptin | **-0.26*** | -0.17 | -0.18 | -0.17 | **-0.60**** | **-0.52**** | 0.13 |
| Matrix correlations are presented as Spearman correlation coefficients (Rho). Significance (boldfaced): *P-value < 0.05, **P-value < 0.001. Abbreviations: 1-RM = one-repetition maximum; CRP = C-reactive protein; HRmax = maximal heart rate; IFNɣ = interferon-gamma; IL = interleukin; TNFα = tumor necrosis factor-alpha; VO2max = maximal oxygen uptake. | | | | | | | |
